# Deciphering the Role of Phytoalexins in Plant-Microorganism Interactions and Human Health

**DOI:** 10.3390/molecules191118033

**Published:** 2014-11-05

**Authors:** Philippe Jeandet, Claire Hébrard, Marie-Alice Deville, Sylvain Cordelier, Stéphan Dorey, Aziz Aziz, Jérôme Crouzet

**Affiliations:** 1 Laboratory of Stress, Defenses and Plant Reproduction, Research Unit “Vines and Wines of Champagne”, UPRES EA 4707, Department of Biology and Biochemistry, Faculty of Sciences, University of Reims, P.O. Box 1039, 51687 Reims cedex 02, France; E-Mails: claire.hebrard@univ-reims.fr (C.H.); sylvain.cordelier@univ-reims.fr (S.C.); stephan.dorey@univ-reims.fr (S.D.); aziz.aziz@univ-reims.fr (A.A.); jerome.crouzet@univ-reims.fr (J.C.); 2 Champagne Deville, 13 rue Carnot, Verzy 51380, France; E-Mail: mdevillegarrick@gmail.com

**Keywords:** phytoalexins, plants, defense mechanisms, microorganisms, biological activity

## Abstract

Phytoalexins are low molecular weight antimicrobial compounds that are produced by plants as a response to biotic and abiotic stresses. As such they take part in an intricate defense system which enables plants to control invading microorganisms. In this review we present the key features of this diverse group of molecules, namely their chemical structures, biosynthesis, regulatory mechanisms, biological activities, metabolism and molecular engineering.

## 1. Phytoalexins: A Global Survey

Phytoalexins take part in an intricate defense system used by plants against pests and pathogens [1,2]. These are low molecular weight antimicrobial compounds both synthesized by and accumulated in plants as a response to biotic and abiotic stresses. The concept of phytoalexins was first introduced over 70 years ago by Müller and Börger [3] after observing that infection of potato tubers with a strain of *Phytophthora infestans* capable of initiating hypersensitive reactions, significantly inhibited the effect of a subsequent infection with another strain of *P. infestans*. This inhibition was linked to a “principle” produced by the plant cells reacting hypersensitively that they named *phytoalexin* [4].

Most of what is known about phytoalexins derives from extensive work on a limited number of plant families: Leguminosae or Fabaceae and Solanaceae [5,6], on one hand, and investigations on one or a few species within other plant families, namely Amaryllidaceae, Euphorbiaceae, Orchidaceae, Chenopodiaceae, Compositae, Convolvulaceae, Ginkgoaceae, Poaceae, Linaceae, Moraceae, Orchidaceae, Piperaceae, Rosaceae, Rutaceae and Umbelliferae on the other hand [7]. More intensive studies recently focused on phytoalexins from plant families of significant economic importance: Poaceae (maize and rice) [8], Vitaceae [9,10] and Malvaceae (cotton) [11]. Camalexin, the main phytoalexin from Brassicaceae (Cruciferae) has also been the subject of numerous studies focusing on its biosynthetic pathway and the regulatory networks involved in its production in the model plant *Arabidopsis thaliana* [1,12]. However, the question of the ubiquity of phytoalexins throughout the plant kingdom still remains.

Phytoalexins are restricted to compounds produced from remote precursors, through *de novo* synthesis of enzymes. This peculiarity makes deciphering their biosynthesis and regulation mechanisms very complex [1,2]. Phosphorylation cascades, defense-related marker genes, calcium sensors and elicitors as well as hormone signaling are potentially important regulators for the modulation of phytoalexin production and pathogen resistance. As a corollary, knowledge of the control mechanisms of phytoalexin accumulation has served as the basis for the genetic manipulation of those compounds in engineered plants for enhanced disease resistance [1,13,14].

The question as to whether phytoalexins are active *in vivo* and play a significant role in plant defense mechanisms has long been debated addressing both the actual antimicrobial activity of phytoalexins under the conditions found within plant tissues and their localization around invading organisms [15,16]. These intractable interrogations are indeed crucial to their proposed role as microbial growth regulators in infected plant tissues. Nonetheless there is considerable evidence that these compounds exhibit *in vitro* toxicity across much of the biological spectrum, prokaryotic and eukaryotic.

The nature of the interaction between plants and pathogens largely depends on the ability of the latter to metabolize the phytoalexins to which they are exposed. Engineering of fungal genes responsible for detoxification of phytoalexins in plants has pointed out their role in the interactions between plants and pathogens [17]. In phytopathogenic fungi, ATP-Binding Cassette (ABC) transporters may also extrude plant defense products as well as fungicides. These transporters act as virulence factors providing protection against phytoalexins produced by the host. Many factors thus interplay to affect the outcome of the interaction between plants and pathogens.

It has recently been demonstrated that phytoalexins may also display health-promoting effects in humans. For instance, resveratrol produced by Vitaceae has been acclaimed for its wondrous effects and its wide range of purported healing and preventive powers as a cardioprotective, antitumor, neuroprotective and antioxidant agent as well as an antifungal and antibacterial compound [14] (see Section 8).

Work on phytoalexins has been prolific and the production of these compounds in infected tissues has become one of the most intensively studied mechanisms of disease resistance in plants. This review will focus on some of the main features of phytoalexins:

○ Chemical diversity
○ Main biosynthetic pathways and regulation networks
○ Biological activity against microorganisms
○ Molecular engineering for disease resistance in plants
○ Metabolism/Transport in fungi
○ Role in human health

## 2. Chemical Diversity of Phytoalexins

Most phytoalexins produced by the Leguminosae belong to six isoflavonoid classes: isoflavones, isoflavanones, pterocarpans, pterocarpenes, isoflavans and coumestans (Table 1) ([1] and references therein). Some pterocarpan phytoalexins are especially well known: pisatin, phaseollin, glyceollin, medicarpin and maackiain. Pisatin was the first phytoalexin to be isolated and characterized from garden pea, *Pisum sativum* [18]. Besides these compounds, a small number of legumes also produce non-isoflavonoid phytoalexins such as furanoacetylenes and stilbenes (Table 1). 

**Table 1:** Phytoalexins from different plant families.

Plant Families (in Alphabetical Order)	Types of Phytoalexins/Examples	References
Amaryllidaceae	Flavans	[19]
Brassicaceae (Cruciferae)	Indole phytoalexins/camalexin	[20]
	Sulfur-containing phytoalexins/brassinin	[21]
Chenopodiaceae	Flavanones/betagarin Isoflavones/betavulgarin	[22]
Compositae	Polyacetylenes/safynol	[23]
Convolvulaceae	Furanosesquiterpenes/Ipomeamarone	[24]
Euphorbiaceae	Diterpenes/casbene	[25]
Poaceae	Diterpenoids:Momilactones; Oryzalexins; Zealexins; Phytocassanes; Kauralexins	[8,26]
	Deoxyanthocyanidins/luteolinidin and apigeninidin	[26,27]
	Flavanones/sakuranetin	[1]
	Phenylamides	[28]
Leguminosae	Isoflavones Isoflavanones Isoflavans Coumestans Pterocarpans/pisatin, phaseollin, glyceollin and maiackiain Furanoacetylenes/wyerone Stilbenes/resveratrol Pterocarpens	[1] and references therein
Linaceae	Phenylpropanoids/coniferyl alcohol	[29]
Malvaceae	Terpenoids naphtaldehydes/gossypol	[11]
Moraceae	Furanopterocarpans/moracins A-H	[30]
Orchidaceae	Dihydrophenanthrenes/loroglossol	[31]
Rutaceae	Methylated phenolic compounds/xanthoxylin	[32]
Umbelliferae	Polyacetylenes/falcarinol	[33]
	Phenolics: xanthotoxin	[34]
	6-methoxymellein	[35]
Vitaceae	Stilbenes/resveratrol	[9]
Rosaceae	Biphenyls/auarperin	[36]
	Dibenzofurans/cotonefurans	
Solanaceae	Phenylpropanoid related compounds	[1] and references therein
	Steroid glycoalkaloids	
	Norsequi and sesquiterpenoids	
	Coumarins	
	Polyacetylenic derivatives	

Chen *et al*., describe a series of compounds produced by the genus *Tephrosia*, which belongs to the Leguminosae family, and possesses phytoalexin-like activities [37]. Five main classes of phytoalexins have been reported in Solanaceae: phenylpropanoid-related compounds, steroid glycolalkaloids, norsesqui- and sesquiterpenoids, coumarins and polyacetylenic derivatives (Table 1) ([1] and references therein).

Although considerable work has been done on phytoalexins from the Leguminosae and Solanaceae families, it has been recently overshadowed by the discovery of two new phytoalexin classes from the Poaceae [8,26] and Brassicaceae families [20,21]. The main phytoalexins of Poaceae (rice, maize and sorghum) are represented by members of the labdane-related diterpenoid superfamily (zealexins, kauralexins, momilactones, oryzalexins and phytocassanes) [8,26], flavanones, an unusual group of flavonoid phytoalexins, the 3-deoxyanthocyanidins [27] and phenylamides [28] (Table 1 and references therein). The current knowledge on phytoalexins produced by sorghum (3-deoxy-anthocyanidins like luteolinidin and apigeninidin) and maize (zealexins, kauralexins) has been reviewed previously [26]. Indole compounds such as camalexin and brassinin represent the major phytoalexins from the Brassicaceae family (Table 1 and references therein).

Not unexpectedly, phytoalexins from very diverse plant families are represented by many different chemical classes. Naphtaldehyde compounds such as gossypol and its derivatives constitute the main Malvaceae phytoalexins [11] (Table 1). Antifungal polyacetylenes have been isolated as phytoalexins from the Compositae and Umbelliferae families [23,33]. Furanosesquiterpenes and diterpenes constitute the phytoalexins from the Convolvulaceae and Euphorbiaceae families [24,25]. The majority of phytoalexins found in the following plant families are phenolic compounds: flavans in Amaryllidaceae [19], flavanones and isoflavones from Chenopodiaceae [22], Linaceae phenylpropanoids [29], furanopterocarpans in Moraceae [30], dihydrophenanthrenes from Orchidaceae [31], Rutaceae methylated phenolics [32], biphenyls and dibenzofurans in Rosaceae [36], xanthotoxin and 6-methoxymellein in Umbellifereae [34,35] and finally hydroxystilbenes from Vitaceae (Table 1) [1,9,10].

## 3. Main Biosynthetic Pathways

Various pathways are utilized for producing different phytoalexins. As it is not our goal to describe each of these biosynthetic routes in details, we will simply outline the three most characteristic ones:

(i) The phenylpropanoic-polymalonic acid route
(ii) The methylerythritol phosphate and geranyl-geranyl diphosphate pathway
(iii) The indole phytoalexin pathway

### 3.1. Phytoalexins Deriving from the Phenylpropanoic-Polymalonic Acid Route

**Figure 1:**
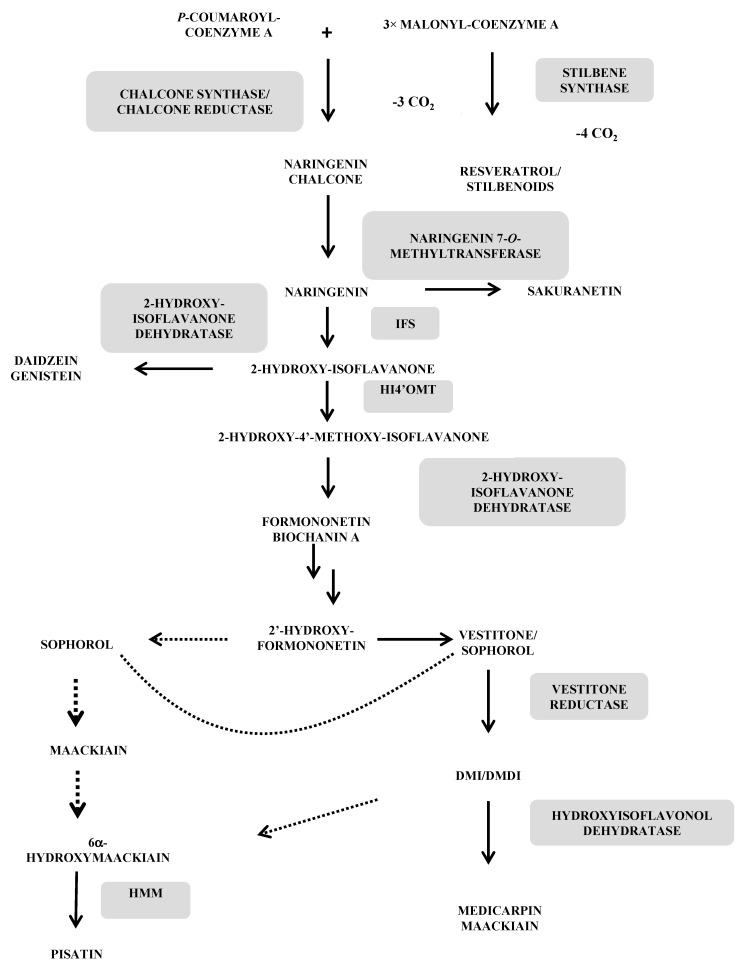
Biosynthetic pathways to the main flavonoid and stilbenoid phytoalexins from the Leguminosae family. (adapted from [10,38,39]). The dashed arrows represent hypothetical steps and the solid arrows denote reactions for which the catalyzing enzymes have been cloned.

All flavonoid phytoalexins (isoflavonoids, isoflavones, pterocarpans, isoflavans, coumestans and arylbenzofurans) as well as stilbene phytoalexins and derivatives (dihydrophenanthrenes) are formed through the universal phenylpropanoic-polymalonic acid pathway. It begins with phenylalanine and the phenylalanine ammonia lyase (PAL) or to a lesser extent with tyrosine and the tyrosine ammonia lyase (TAL). The obtained *para*-coumaric acid is activated in *para*-coumaroyl-CoA by ligation to a coenzyme A by 4-coumaroyl:CoA ligase (C4L). Subsequently, chalcone synthase (CHS) on the one hand and stilbene synthase (STS) on the other hand use this same substrate and condense it with three successive units of malonyl-CoA, leading respectively to the production of naringenin chalcone, the first C15 intermediate in the flavonoid pathway and resveratrol, the precursor of all stilbenes. The possible biosynthetic routes to the main flavonoid and stilbene-like phytoalexins from the Leguminosae family are illustrated in Figure 1 [10,38,39].

### 3.2. Mevalonoid-Derived Phytoalexins

**Figure 2:**
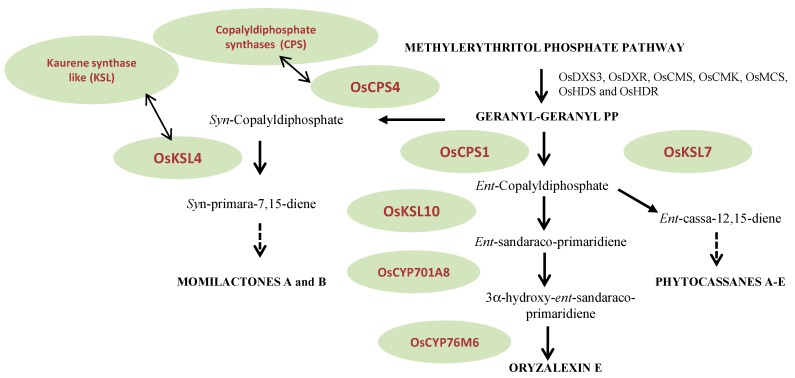
Biosynthetic pathway of diterpenoid phytoalexins.

These phytoalexins are represented by members of the monoterpene, sesquiterpene, carboxylic sesquiterpene and diterpene families. Specific attention will be given to the diterpene phytoalexin class [8]. This assumption has been confirmed by the observed synchronous accumulation of seven MEP pathway gene transcripts (OsDXS3, OsDXR, OsCMS, OsCMK, OsMCS, OsHDS and OsHDR) in elicitor-induced rice (*Oryza sativa*) cells and the next steps of this biosynthesis are predicted to occur in plastids. Diterpenoids result from the subsequent action of diverse enzymes using GGDP as the starting block. Class II diterpene cyclases named copalyldiphosphate synthases (CPS) are the first to act on GGDP catalyzing the initial cyclization of the latter to copalyldiphosphate (CDP). CDP is the required substrate for class I diterpene synthases named kaurene synthase like (KSL). Sequential action of CPS and KSL produces the olefin precursors of the main diterpene phytoalexin families [8]. Stereochemically differentiated isomers are used subsequently by KSL: the *ent*-CDP in the biosynthesis of phytocassanes A-E and oryzalexins A-F and the *syn*-CDP in the construction of momilactones A and B (Figure 2). Further additions of oxygen in the formation of oryzalexins, momilactones and phytocassanes require a series of cytochrome P450 (CYPs) (Figure 2).

### 3.3. Indole Phytoalexins

**Figure 3:**
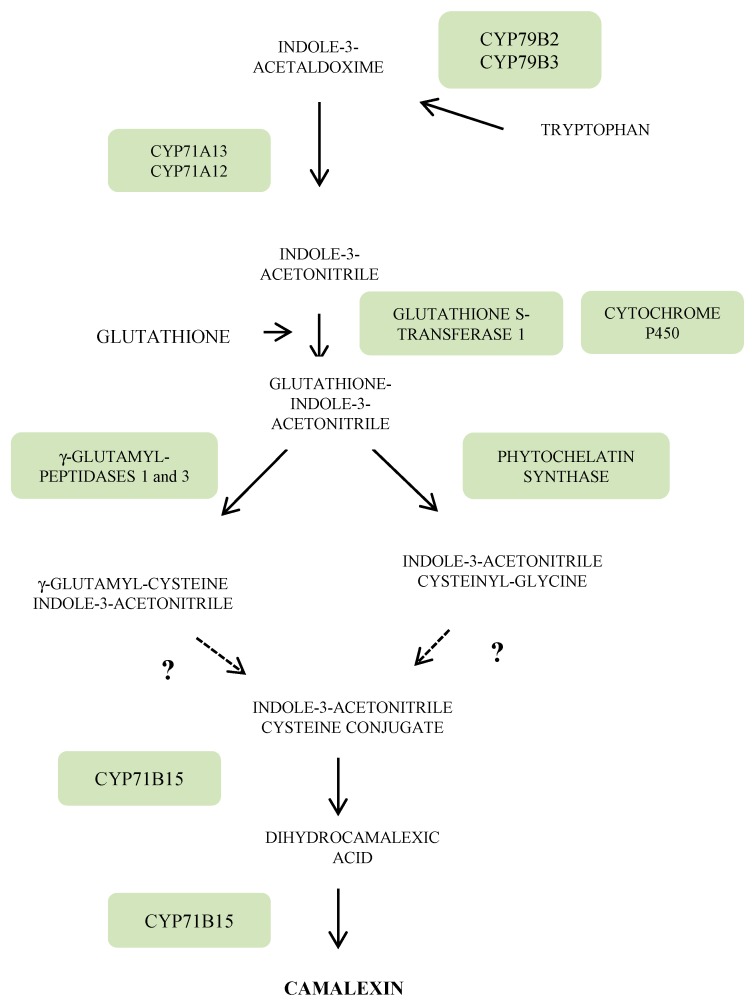
Biosynthetic pathway from tryptophan to camalexin (adapted from [2]).

Specific attention will be paid in this section to camalexin, the major phytoalexin of Arabidopsis. The indolic ring of camalexin is derived from tryptophan (Trp) which in turn arises from chorismate (Figure 3). The first step in the route from Trp to camalexin is under the control of two cytochrome P450 homologues CYP79B2 and CYP79B3, leading to indole-3-acetaldoxime. The latter is then transformed into indole-3-acetonitrile (IAN) via the cytochrome P450, CYP71A13. Subsequent conjugation of IAN with glutathione is performed by the combined action of a glutathione-S-transferase and most likely a cytochrome P450. The IAN glutathionyl derivative is then converted into IAN cysteinyl-glycine via a phytochelatin synthase or into γ-glutamyl-cysteine IAN through the action of two γ-glutamyltranspeptidases 1 and 3 [2]. Both intermediates lead to the IAN cysteine conjugate. The last steps of this biosynthesis pathway are under the control of a *CYP71B15* (*PHYTOALEXIN DEFICIENT 3*, *PAD 3*) gene encoding a multifunctional enzyme which forms camalexin via dihydrocamalexic acid (Figure 3).

## 4. Regulation Networks

Phytoalexin biosynthesis is up- or downregulated by expression of many endogenous molecules such as phytohormones (jasmonic acid, salicylic acid, ethylene, auxins, abscisic acid, cytokinins and to a lesser extent gibberellins), transcriptional regulators, defense-related genes, phosphorylation relays and cascades [1,2].

Regulatory mechanisms of phytoalexin biosynthesis also depend on the nature of the infecting pathogen as well as the nature of the induced phytoalexin itself. For example, in the Arabidopsis-*Alternaria brassicicola* interaction, accumulation of camalexin was reported to be independent from jasmonic acid (JA) [40,41] though JA was involved in the regulatory signaling pathways of this phytoalexin in Arabidopsis plants challenged with the fungal pathogen *Botrytis cinerea* [42]. Similarly, existence of JA-dependent and independent pathways in the modulation of diterpenoid phytoalexins in the interaction between rice and the fungal agent *Magnaporthe oryzae* was clearly evidenced by the use of rice mutants [43]. These mutants lacking a functional allene oxide cyclase required for JA production were impaired in momilactone accumulation upon fungal infection whereas phytocassane production was not altered. 

Besides, regulation of camalexin production in *Arabidopsis* is controlled by either salicylic acid (SA)-independent [44,45] or SA-dependent signaling pathways [46]. Indeed, biosynthesis of this phytoalexin was also found to be lower in SA-induction deficient mutants of *Arabidopsis* with impaired production of ethylene upon bacterial infection by *Pseudomonas syringae* [47].

Other phytohormones have been involved in the regulatory mechanisms of phytoalexin biosynthesis. Auxins and abscisic acid (ABA) generally appear to negatively regulate phytoalexin production [1]. Suppression of auxin signaling has recently been shown to increase the resistance of Arabidopsis to biotrophic pathogens and to redirect phytoalexin metabolism [48]. The biosynthesis of numerous phytoalexins is downregulated by ABA. For example, synthesis of kievitone in bean [49], synthesis of glyceollin in soybean [50,51] and production of rishitin and lubimin in potato [52] are all decreased by ABA. Tobacco mutants deficient in ABA exhibit twice as much capsidiol as wild-type plants [53]. In contrast, cytokinin overexpression was shown to enhance resistance of tobacco to *P. syringae* [54]. This increased resistance correlated well with the up-regulated synthesis of two phytoalexins, capsidiol and scopoletin. 

Mitogen-Activated Protein Kinases (MAPKs) have been involved in the induction of camalexin accumulation in Arabidopsis plants upon treatment with Microbe-Associated Molecular Patterns (MAMPs) [12]. Specifically two MAP kinases, MPK3 and MPK6 take part in the up-regulation of numerous enzymes of the camalexin biosynthetic route. For example, expression of the CYP71B15 gene, which encodes the multifunctional enzyme acting at the end of the pathway showed a 400-fold increase upon overexpression of these two MAPKs [12]. In Arabidopsis *mpk3*/*mpk6* double mutants, camalexin production was completely abolished concomitantly with an increased susceptibility to *B. cinerea*.

Protein phosphorylation-induced phytoalexin production is also under the control of cell calcium transfers which in turn are decoded and transmitted by a toolkit of calcium binding proteins [1]. Several families of calcium sensors are indeed involved in the phytoalexin regulation networks. For instance, overexpression of two genes encoding a calcineurin B-like protein-interacting protein kinase in rice was found to induce two phytoalexin classes, phytocassanes and momilactones upon MAMP treatment [55].

Other regulators of phytoalexin biosynthesis have been identified [1]. Overexpression of Rac proteins in rice induced disease resistance to bacterial blight together with a 19- to 180-fold increase in the accumulation of the rice phytoalexin momilactone A [56]. Production of this phytoalexin is also controlled by selenium-binding protein homologues as shown in the interaction between rice and both the rice blast fungus and the rice bacterial blight [57]. Overexpression of microbial virulence factors belonging to the Nep1-like protein family in *Arabidopsis* was associated with a strong transcriptional activation of genes involved in the camalexin route [58]. Various sugars (sucrose, glucose and fructose) acting as endogenous signals, have been reported to regulate the biosynthesis and accumulation of some phytoalexins [59]. Finally, overexpression of non-expressor of pathogenesis-related genes-1 which play a critical role in the systemic acquired resistance was reported to induce the biosynthesis of the cotton phytoalexin gossypol [60]. Knowledge of the regulatory mechanisms of phytoalexin biosynthesis thus paves the way for metabolic engineering of plants for disease resistance (see Section 6).

## 5. Biological Activity against Microorganisms

Are phytoalexins biologically active compounds? Do phytoalexins show antibacterial activities? Over 70 years after their discovery, the actual role of phytoalexins in plant defense mechanisms is still debated. Phytoalexins are considerably less toxic than chemical fungicides. Lack of activity of isoflavonoid phytoalexins was indeed reported in comparison to classic fungicides like benomyl and mancozeb [61]. Effective doses of phytoalexins generally fall within orders of magnitude 10^−5^ to 10^−4^ M [62,63]. Phytoalexin fungitoxicity is clearly evidenced by the inhibition of germ-tube elongation, radial mycelial growth and/or mycelia dry weight increase, as best illustrated by the action of resveratrol on *B. cinerea*, the causal agent for gray mold in grapevine [63,64]. Phytoalexin antifungal activity can considerably vary from one compound to another. For example, Hasegawa *et al*., show that the rice phytoalexin sakuranetin displays a higher activity against the blast fungus than does another rice phytoalexin, momilactone A, both *in vivo* and *in vitro* [65].

Phytoalexins may also exert some effects on the cytological, morphological and physiological characteristics of fungal cells. The activity of four phytoalexins from the Solanaceae family (rishitin, phytuberin, anhydro-β-rotunol and solavetivone) on three *Phytophthora* species resulted in loss of motility of the zoospores, rounding-up of the cells associated with some level of swelling, cytoplasmic granulation and bursting of the cell membrane [66]. The two latter are very general features of the action of phytoalexins on fungal cells ([63,64,67] and references therein). The extensive membrane damage occurring after fungal exposure to phytoalexins is reflected in substantial leakage of electrolytes and metabolites [68]. However, it has been observed that despite the presence of wyerone acid or resveratrol, surviving *B. cinerea* fungal cells could produce secondary and to a lesser extent tertiary germ tubes suggesting that some sort of escape from phytoalexin damage could take place [63,69]. Asymetric growth of the germ tube resulting in the production of “curved-germ tubes” has also been observed in *B. cinerea* conidia treated with sub-lethal doses of resveratrol [63]. This cytological abnormality suggests that stilbenic compounds may interact with tubulin polymerization, the mode of action of many synthetic fungicides and anticancer agents [70]. Moreover, phytoalexins may affect glucose uptake by fungal cells as reported in the interactions between phaseollin or kievitone/and *Rhizoctonia solani* [68]. Observations of *B. cinerea* conidia showed a complete disorganization of mitochondria and disruption of the plasma membrane upon treatment with the stilbene phytoalexins, resveratrol and pterostilbene [63,64,67]. Pterostilbene especially led to a rapid and complete cessation of respiration in *B. cinerea* conidia which can be explained by its activity as an uncoupling agent of electron transport and phosphorylation [67]. Camalexin has recently been involved in the induction of fungal apoptotic programmed cell death in *B. cinerea* [71]. The efficaciousness *in vivo* of some phytoalexins, namely the coumarin phytoalexin, scopoletin on the reduction of green mold symptoms caused by *Penicillium digitatum* on oranges was shown [72]. In the same way, phenolic phytoalexins (resveratrol, scopoletin, scoparone and umbelliferone) were shown to significantly inhibit the growth of *Penicillium expansum* and patulin accumulation in apples [73]. To increase the fungitoxicity of phytoalexins, design and synthesis of more active phytoalexin derivatives is needed [74,75].

Beside their antifungal activity, phytoalexins possess some antibacterial activity. Rishitin for instance decreased the viability of cells of *Erwinia atroseptica* by around 100% at a dose of 360 μg/L [76]. Resveratrol also exerts some activity against numerous bacteria affecting humans: *Chlamydia*, *Helicobacter*, *Staphylococcus*, *Enterococcus*, *Pseudomonas* and *Neisseria* ([14] and references therein). It is thus clear that phytoalexins exhibit toxicity across much of the biological spectrum, prokaryotic and eukaryotic.

## 6. Engineering of Phytoalexins and Role in Plant Defense Mechanisms

Gain- or loss-of-function genetic approaches addressing phytoalexin production for disease resistance have provided direct and indirect proofs of their implication in plant/microorganism interactions. Relatively simple genetic constructs involving the introduction of a single gene in plants are required in the case of the grapevine phytoalexin resveratrol, synthesis of which is controlled by the stilbene synthase (STS). The first report of increased disease resistance resulting from foreign phytoalexin expression in a novel plant was brought by the group of Kindl with the transfer of two grapevine STS genes (*Vst 1* and *Vst 2*) into tobacco [77]. Introduction of these two genes was shown to confer higher resistance to *B. cinerea*. From that point, a number of transformations were then completed in alfalfa, rice, barley, wheat, tomato, papaya and Arabidopsis using the same STS genes or STS genes from other plant origins, conferring resistance to various pathogens [1]. All these results clearly showed that phytoalexins could act as determinant factors in the expression of the defense mechanisms of plants against phytopathogenic microorganisms though there are rare examples of STS overexpression not being associated with disease resistance [1].

Following the works on stilbene phytoalexins, other genetic transformations were achieved with other phytoalexin genes. Surprisingly, engineering phytoalexins seems to have been limited to exploiting only a few phytoalexin biosynthetic genes. This has mainly concerned the genetic manipulation of phytoalexin glycosylation by the use of a tobacco glucosyltransferase acting on scopoletin [78]. Overexpression of the isoflavonoid-7-*O-methyltransferase* in alfalfa, an enzyme with a crucial role in the biosynthesis of the phytoalexin maiackiain, was also linked to an increased resistance of that plant to *Phoma medicaginis* [79]. Transformation of soybean hairy roots with both the peanut resveratrol synthase 3 *AhRS3* gene and resveratrol-*O-methyltransferase ROMT* gene catalyzing the transformation of resveratrol to pterostilbene [80] resulted in the resistance of that plant to *Rhizoctonia solani* [81]. In many cases, engineering the entire phytoalexin biosynthetic pathway is not feasible and the problem researchers are facing is to choose the right enzyme catalyzing the limitant step of this pathway.

Loss-of-function genetic approaches clearly evidenced the role played by phytoalexins in plant-microorganism interactions. In almost all experiments, mutants impaired in phytoalexin production showed increased susceptibility to pathogens. Reduced amounts of pisatin in hairy roots of pea transformed with antisense 6-α-hydroxymaiackiain-3-*O-*methyltransferase were associated with a decreased resistance to the fungal pathogen *Nectria haematococca* [17]. RNAi silencing of isoflavone synthase or chalcone reductase in soybean suppressed by 90% the accumulation of daidzein and glyceollin as well as disease resistance to *P. sojae* [82]. Loss-of-function alleles of the *yellow seed1* gene encoding CHS, chalcone isomerase, dihydroflavonol reductase and flavonoid-3'-hydroxylase induced deficiency in the accumulation of 3-deoxyanthocyanidin associated with severe symptoms of the anthracnose disease in sorghum [83]. Effect of the *PHYTOALEXIN DEFICIENT* mutation on camalexin in Arabidopsis was found to be dependent on the infecting pathogen. This mutation was not associated with increased susceptibility to *P. syringae*, *Perenospora parasitica*, *Erysiphae oronti* and *B. cinerea* though it markedly affected its susceptibilty to *A. brassicicola* ([1] and references therein). Finally loss-of-function genetic approaches have underlined the role of phytoalexin glycosylation in plant-pathogen interactions. Transgenic tobacco leaves downregulated for a tobacco specific phenylpropanoid glucosyltransferase saw their scopolin content decreased by 70% to 75% associated with a 63% increase in TMV lesion surfaces [84].

Indirect modulation of phytoalexin levels through manipulation of hormone signaling, phosphorylation cascades or defense-related marker genes also demonstrated the role of phytoalexins in plant defense mechanisms. For instance cytokinin overexpression in tobacco led to increased resistance to *P. syringae* which strongly correlated with up-regulated synthesis of two phytoalexins, capsidiol and scopoletin [54]. Mutations in two MAP kinases MPK3 and MPK6 impaired camalexin production and disease resistance to *B. cinerea* in Arabidopsis [12].

Though phytoalexin engineering seems to have been limited to exploiting only a few genes mainly stilbene and isoflavonoid ones, indirect modulation of phytoalexin accumulation employing transcriptional regulators or components of upstream regulatory pathways becomes a useful approach to improve plant disease resistance [1].

## 7. Fungal Metabolism and Transporters

The interaction between a plant and its pathogen can be envisaged as a balance between phytoalexin production by the host and phytoalexin metabolism or inactivation via transporters (mainly ATP Binding Cassette, ABC transporters) by the second actor of this interaction. Modification of any of the factors contributing to this balance could modify the outcome of the interaction. We will further see that in phytopathogenic fungi, ABC transporters act as virulence factors, conferring protection against defense compounds produced by the host. In several plant-fungus interactions, it has become evident that the ability to weaken or neutralize the effects of phytoalexins is one of the essential determinants of fungal/host coupling. Detoxification processes of phytoalexins by fungi are far from being clearly understood [85].

It is rather difficult to derive any comprehensive generalizations from the existing data on phytoalexin metabolism *per se*. The known catabolic pathways of phytoalexins by fungi may involve monoxygenation, reduction, hydration, oxidation, oxidative dimerization, glycosylation and demethylation reactions. Since most phytoalexins are lipophilic compounds that efficiently penetrate cell membrane structures, phytoalexin metabolism usually involves their conversion to more polar products. Creation of new hydroxyl groups by oxygenation, demethylation, reduction of aldehydes and ketones or hydration of double bonds as well as glycosylation increase the degree of polarity of phytoalexins. Detoxification of the cruciferous phytoalexins, brassinin, 1-methoxybrassinin and cyclobrassinin, by the stem rot fungus *Sclerotinia sclerotiorum* indeed requires a brassinin glucosyltransferase [86]. Methylated phytoalexins are well known to be more fungitoxic than the non-methylated ones owing to the fact that phytoalexin methylation enhances their lipophilic character. Moreover the presence of methylated groups or any other electron-attracting groups on the aromatic ring of some phytoalexins plays an important role in the formation of charge transfer complexes, favoring contact and affinity with (membrane) proteins and acting as uncoupling agents of electron transport and photophosphorylation. A cytochrome P450 pisatin demethylase transforming pisatin into 6-α-hydroxymaiackiain and 3-hydroxymaackiain-isoflavan was characterized from the fungal pathogen *Nectria haematococca* [87]. Interestingly, fungal isolates with the highest pisatin demethylating activity were shown to be the most virulent on pea [87]. In addition, overexpression of this pisatin demethylating activity in hairy roots of pea resulted in reduced amounts of this phytoalexin in the plant tissues upon infection by *N. haematococca* with a correlated decreased resistance to this pathogen [17]. Two hydratases, a kievitone hydratase from *Fusarium solani* [88] and one inducible hydrolase of *Leptosphaeria maculans* acting on brassinin [89] were implicated in the detoxification process of these two phytoalexins.

Oxidation and oxidative dimerization processes can also take place in the metabolism of phytoalexins. A brassinin detoxifying oxidase with a molecular mass of 57 kDa has been characterized and purified from the blackleg fungus *L. maculans*. This oxidase transforms the cruciferous phytoalexin brassinin into the less fungitoxic compound indol-3-carboxaldehyde [90]. Interestingly, this pathogen was unable to metabolize camalexin, another major phytoalexin from crucifers, conferring this plant family protection against *L. maculans*. Simple stilbenes produced by members of the Vitaceae family may undergo oxidative dimerization by a laccase-like stilbene oxidase from *B. cinerea* with a molecular mass of 32 kDa [91]. This process involves the 4'-hydroxyphenyl group of one resveratrol unit leading to a dehydrodimer with a dihydrobenzofuran structure named δ-viniferin [92,93]. Importantly, *B. cinerea* isolates possessing the highest oxidative activity were found to be the most virulent on grapevine [94]. Very recently, the stilbene-type phytoalexin astringin produced by Norway spruce in the interaction with the bark beetle (*Ips* spp.) and its fungal associate, *Ceratocystis polonica* was shown to undergo metabolism by the latter [95]. *C. polonica* converted astringin to ring-opened lactones, aglycones and dehydrodimers *in vitro*. In this study, the virulence of the fungal pathogen on Norway spruce correlated well with differential usage of the various pathways for stilbene biotransformation.

Phytopathogenic fungi evolved mechanisms of insensitivity or resistance to protect themselves against phytoalexins. One of them involves extruding toxic compounds out of the cell through transporters, conferring them protection against plant defense products. In fungi, the role of ATP-binding cassette (ABC) transporters in the efflux of natural and synthetic toxicants is well known [96,97,98]. In addition, several genes encoding ABC transporters have been shown to be involved in fungal virulence on plant hosts [99,100,101,102,103,104]. A number of phytoalexins and other toxic compounds induced expression of these fungal transporters [99,100,105,106,107]. However, only a few studies have demonstrated the ability of these transporters to confer tolerance to a known phytoalexin. In *B. cinerea*, the BcatrB ABC transporter has been shown to be a virulence factor that increases tolerance of the pathogen towards phytoalexins. Indeed, *BcatrB* replacement mutants revealed increased sensitivity to resveratrol and reduced virulence on grapevine leaves [100]. Moreover, a *B. cinerea* strain lacking functional BcatrB was more sensitive to camalexin *in vitro* and less virulent on *A. thaliana* wild-type plants, but was fully virulent on camalexin-deficient *A. thaliana* mutants [103]. In the same way, ABC transporters related to *BcatrB*, such as GpABC1 from *Gibberella pulicaris*, act as virulence factors on potato. GpABC1 provides tolerance to rishitin, while a *GpABC1* mutant is essentially non-pathogenic [101]. In *N. haematococca*, the *NhABC1* gene is induced after treatment with pisatin *in vitro* and during infection of pea plants. Mutation in *NhABC1* gene rendered the fungus even more sensitive to pisatin and led to lower pathogenicity on pea, indicating that *NhABC1* contributes to the tolerance to pisatin and acts as a virulence factor [102]. The substrate range of ABC transporters can vary from a single compound to a wide spectrum of molecules with no identified common feature. BcatrB from *B. cinerea* has a wide substrate range, comprising mainly aromatic compounds [100,103,107,108,109] such as the phytoalexins eugenol, resveratrol and camalexin, the fungicides fenpiclonil and fludioxonil, as well as the antibiotics phenazine-1-carboxylic acid and phenazine-1-carboxamide. The closest identified homologue of BcatrB, AtrB from *Aspergillus nidulans*, shows a similar function in multidrug resistance [110]. Two other homologues with similar substrate ranges are MgAtr5 from *Mycosphaerella graminicola* [111] and PMR5 from *Penicillium digitatum* [112], indicating that other homologues of BcatrB may function also in multidrug resistance and pathogenesis. Taken together, it becomes evident that ABC transporters can be essential for the development of phytopathogenic fungi, providing protection against phytoalexins produced by the host plant and acting as virulence factors.

## 8. Role of Phytoalexins in Human Health

Phytoalexins may display health-promoting effects in humans. A few of them have been reported to exert antioxidant, anticarcinogenic and cardiovascular protective activities. Maslinic acid, a natural phytoalexin-type triterpene from olives exerts a wide range of biological activities as an antitumor, antidiabetic, neuroprotective, cardioprotective, antiparasitic and growth-stimulating agent, providing evidence of the potential of this molecule as a nutraceutical [113]. Health benefit properties of Brassicaceae were attributed in part to their phytoalexins, camalexin and related indolic compounds [114]. Camalexin namely is able to induce apoptosis in prostate cancer cells [115]. 3-deoxyanthocyanidins, flavonoid phytoalexins produced by members of the Poaceae family, are helpful in reducing the incidence of gastro-intestinal cancer [116]. Moreover, other indolic phytoalexins such as brassinin and its derivative, homobrassinin, show marked antiproliferative activities in human colorectal cancer cells *in vitro* [117]. The most promising results in this area were obtained with resveratrol, the phytoalexin from grapevine. This compound is indeed considered to be an antiproliferative agent exerting antitumor activity either as a cytostatic or a cytotoxic agent in various cancers [118]. The first report of the cancer chemoprotective activity of a phytoalexin is the study of the group of Pezzuto [119]. This pioneering work was then confirmed on many other human cancer models. The most frequently described mode of antitumor action for phytoalexins concerns apoptosis which may be via the inhibition of antiapoptotic molecules such as survivin [120] or, for instance as reported in this issue, alterations of expression and activity of lysosomal protease cathepsin D [115].

Resveratrol exerts antitumor activities *in vivo*, namely in skin cancers by topical applications. It does not seem to be very effective in inhibiting leukemia despite displaying antileukemic activity *in vitro* and shows anticancer effects in experimentally-induced breast cancers only at high doses. Resveratrol presents some anticancer activities in hepatoma, lung carcinoma and intestinal tumors ([14] and references therein). However lack of efficacy of natural phytoalexins in reducing tumors has led to a number of investigations regarding the design and synthesis of more potent anticancer derivatives of known phytoalexins such as brassinin [121], methoxybrassinol [122] and resveratrol [123].

There are also several studies providing evidence of the cardioprotective activity of phytoalexins such as indoles and stilbenes [14,114]. Resveratrol namely was proven to inhibit LDL peroxidation in *ex vivo* rat heart studies, to have a potent role in preventing atherosclerosis and to block platelet aggregation from high-cholesterol-fed rabbits ([14] and references therein). Besides, this compound has an effect in neurological diseases such as cerebral ischemia, Parkinson’s disease, pain and cognitive impairment in rats, spinal cord lesion in rabbits and finally brain edema and tumors in human cells ([14] and references therein). Interestingly, additional studies have also demonstrated that resveratrol increases lifespan in lower organisms (yeast, metazoans) and higher organisms through the activation of the sirtuin proteins [124,125]. Resveratrol’s mechanisms of action are likely to be pleitropic and mediated by the interaction of this compound with key signaling proteins controlling cellular calcium homeostasis [126]. Interestingly, quercetin and umbelliferone were also reported to reduce mycotoxin accumulation in apple fruits by *P. expansum* by down-regulating relative expression of genes encoding patulin biosynthesis [127].

Some other phytoalexins like the steroid glycoalkaloids from potato or the dimeric sesquiterpene gossypol from cotton display a certain level of toxicity for humans explaining the crucial interest in engineering those plants for abolishing production of these undesirable compounds [11,128].

## 9. Concluding Remarks

Works on phytoalexins from diverse chemical families have generated a lot of data regarding basic aspects of plant defenses and their regulatory mechanisms. As a result, engineering of phytoalexins has arisen as a new area in the development of useful approaches to disease control. Nonetheless, while a variety of genetic transfers were carried out in order to investigate the potential of stilbene and flavonoid phytoalexin biosynthetic genes in conferring disease resistance, strategies focusing on the other phytoalexin chemical families did not [1].

Some studies have attempted to determine the actual concentration and the nature of phytoalexins directly in plant tissues in response to invading microorganisms using spectroscopic methods [129,130]. However, our general knowledge remains limited by the difficulty to analyse the events occurring under natural conditions between the plant and the pathogen.

On the other hand, the potential value of several phytoalexins on a therapeutic point of view has made their large-scale production a necessity. Engineering yeast and bacteria, may represent valuable means for the production of phytoalexins at an industrial scale [14,131]. However, their tailoring is needed as they do not possess the genes encoding phytoalexin biosynthesis. Another approach is large-scale production of phytoalexins using plant cell suspensions in bioreactors. Some experiments are underway to optimize stilbene phytoalexin production in bioreactors [132,133].

Although considerable work has already been done on phytoalexins, the ways in which they act against microorganisms and the mechanisms the latter have developed to counteract their action are still poorly understood keeping this subject an active field of research even after over 70 years.

## Abbreviation

IFS: 2-hydroxy isoflavanone synthase;
DMI: 7,2'-dihydroxy-4'-methoxy-isoflavanol;
DMDI: 7,2'-dihydroxy-4',5'-methylenedioxy-isoflavanol;
HMM: 6α-hydroxymaackiain 3-*O*-methyltransferase
HI4'OMT: SAM: 2,7,4'-trihydroxy-isoflavanone 4'-*O*-methyltransferase;
STS: stilbene synthase;
CHS: chalcone synthase.
